# Economic evaluation of personalised versus conventional risk assessment for women who have undergone testing for hereditary breast and ovarian cancer genes: a modelling study

**DOI:** 10.1136/jmg-2024-109948

**Published:** 2025-04-10

**Authors:** Qin Xi, Nichola Fennell, Stephanie Archer, Marc Tischkowitz, Antonis C Antoniou, Stephen Morris

**Affiliations:** 1 Department of Public Health and Primary Care, University of Cambridge, Cambridge, UK; 2 Department of Medical Genetics, University of Cambridge, Cambridge, UK

**Keywords:** genetic testing, economics, costs and cost analysis

## Abstract

**Background:**

The management of women with germline pathogenic variants (GPVs) in breast (BC) and ovarian cancer (OC) susceptibility genes is focused on surveillance and risk-reducing surgery/medication. Most women are assigned an average range of risk and treated accordingly, but it is possible to personalise this. Here, we explore the economic impact of risk personalisation.

**Method:**

We compared two strategies for risk stratification for female participants: conventional risk assessment (CRA), which only involves information from genetic testing and personalised risk assessment (PRA), using genetic and non-genetic risk modifiers. Three different versions of PRA were compared, which were combinations of polygenic risk score and questionnaire-based factors. A patient-level Markov model was designed to estimate the overall National Health Service cost and quality-adjusted life years (QALYs) after risk assessment. Results were given for 20 different groups of women based on their GPV status and family history.

**Results:**

Across the 20 scenarios, the results showed that PRA was cost-effective compared with CRA using a £20 000 per QALY threshold in women with a GPV in *PALB2* who have OC or BC+OC family history, and women with a GPV in *ATM*, *CHEK2*, *RAD51C* or *RAD51D*. For women with a GPV in *BRCA1* or *BRCA2*, women with no pathogenic variant and women with a GPV in *PALB2* who have unknown family history or BC family history, CRA was more cost-effective. PRA was cost-effective compared with CRA in specific situations predominantly associated with moderate-risk BC GPVs (*RAD51C*/*RAD51D*/*CHEK2*/*ATM*), while CRA was cost-effective compared with PRA predominantly with high-risk BC GPVs (*BRCA1*/*BRCA2*/*PALB2*).

**Conclusion:**

PRA was cost-effective in specific situations compared with CRA in the UK for assessment of women with or without GPVs in BC and OC susceptibility genes.

**WHAT IS ALREADY KNOWN ON THIS TOPIC**

- Risk personalisation for women who have undergone testing for hereditary breast and ovarian cancer genes has been studied under clinical trials, but no economic evaluations have been conducted.

**WHAT THIS STUDY ADDS**

- From this study, we learnt that personalised risk assessment was cost-effective compared with conventional risk assessment in the UK for assessment of women with or without germline pathogenic variants in breast and ovarian cancer susceptibility genes in certain situations.

**HOW THIS STUDY MIGHT AFFECT RESEARCH, PRACTICE OR POLICY**

- This might guide the clinical implementation of personalised risk assessment.

## Introduction

 Breast cancer (BC) is the most commonly diagnosed cancer and a leading cause of death among women in the UK and worldwide.^1^ Annually, around 55 000 women are diagnosed with BC and 11 500 deaths occurred due to BC in the UK in 2011.^2^ Ovarian cancer (OC) is the sixth most common cancer among females in the UK—around 7500 women are diagnosed with OC each year and the annual number of deaths is estimated to be over 4100.^3^ There are a number of options available for the prevention of BC and OC, and early detection of BC. Germline genetic testing using Hereditary Breast and Ovarian Cancer (HBOC) gene panels can be used to identify individuals at a higher risk than those in the general population.

Around 3% of BCs and 10% of OCs are associated with *BRCA1/BRCA2* germline pathogenic variants (GPVs), and 5%–10% of BCs are associated with GPVs in *BRCA1*, *BRCA2*, *PALB2*, *CHEK2*, *ATM*, *RAD51C, RAD51D* or *BARD1*.^4^,^6^ Furthermore, polygenic risk scores (PRS) derived from population-based studies have been shown to modify BC and OC risk for those with GPVs in *BRCA1* and *BRCA2*.^7 8^ Mammographic density (MD) and questionnaire-based factors (QRF) including height, body mass index, parity, age at first birth, age at menarche, age at menopause, use of oral contraceptive, use of hormone replacement therapy and alcohol intake are also associated with BC and OC risk.^9^ Joint consideration of GPVs, PRS, QRFs and MD can lead to more personalised risk assessment (PRA) methods and could potentially better stratify individuals at risk of these cancers.^9 10^

Individuals identified as being at higher risk for BC may undergo preventative interventions.^11^ Risk-reducing mastectomy (RRM) reduces the risk of BC by over 90%.^12^ Other options include risk-reducing medication as well as surveillance methods such as regular MRI or mammography screening.^9 11 13^ Similarly, risk-reducing salpingo-oophorectomy (RRSO) could reduce the risk of OC by over 90%.^14^ There is no effective surveillance strategy for OC currently.^15^

The implementation of interventions and the incidence of cancer impact pecuniary and non-pecuniary costs both on the patient and the health system.^16^ Thus, economic evaluation of different risk assessment approaches, including comprehensive risk-based information for predicting BC and OC risk for women with GPVs in cancer susceptibility genes is critical, as it could identify the most cost-effective way of measuring risk in at-risk populations and identifying populations most at need of taking action.

There have been several economic analyses in different healthcare systems that have evaluated targeted population genetic testing for HBOC, including *BRCA1/2* mutation testing, and testing for multigene panels like 7-gene or 14-gene panels (eg, Manchanda *et al* in the UK^11^; Manchanda *et al* in the UK, the USA, the Netherlands, China, India and Brazil^17^; Guzauskas *et al* in the USA^18^). Additionally, there has been a study on the prevention strategies for HBOC among GPV carriers (eg, Wei *et al* in the UK^19^). Evaluations of risk-stratified screening strategies for BC have also been conducted in various countries (eg, Pashayan *et al* in the UK^20^; Wong *et al* in Singapore^21^; Mital *et al* in the USA^22^). These studies showed that such tests were largely cost-effective. However, no study has so far compared PRA approach in women with GPVs in HBOC genes based on combinations of PRS, QRF and MD information and the conventional risk assessment (CRA) approach based on traditional genetic testing and average cancer risks for counselling.

This study aims to assess the cost-effectiveness of CRA in comparison with different PRA options for BC and OC risk stratification. The investigation focuses on women who have undergone presymptomatic testing in *BRCA1*, *BRCA2*, *PALB2*, *CHEK2*, *ATM, RAD51C* and *RAD51D*. Specifically, we compared: (1) CRA—genetic testing followed by prevention and surveillance interventions based on average risk of GPV carriers and (2) PRA—genetic testing plus personalised assessment with different combinations of QRF and PRS, followed by prevention and surveillance interventions based on individualised risk estimation.

## Methods

A patient-level simulation model was used to simulate the process starting in year 2023 for women aged 25 years until death or they reach the age of 70 years. The cost calculations were performed from a UK National Health Service (NHS) perspective, including risk assessment costs, cancer prevention and surveillance costs and cancer diagnosis and treatment costs. The health outcomes were measured in terms of quality-adjusted life years (QALY).^23^ Future costs and utilities were discounted at a 3.5% annual rate.^23^

### Model structure

We developed a patient-level Markov model. Participants were classified in high-risk, moderate-risk or near-population-risk groups for either BC or OC: high-risk for BC and OC (group HH—*BRCA1*, *BRCA2*), high-risk for BC and near-population-risk for OC (group HN—*PALB2*), moderate-risk for BC and high-risk for OC (group MH—*RAD51C, RAD51D*) or moderate-risk for BC and near-population-risk for OC (group MN—*ATM, CHEK2*), or women with no GPV. Participants were assumed to enter the cohorts with unknown family history, mother diagnosed with BC at age 50 years (BC FHx), mother diagnosed with OC at age 50 years (OC FHx) or mother diagnosed with both BC and OC at age 50 years (BCOC FHx). Separate models were performed for women with different GPVs and family histories; with five GPV groups and four family history groups, we estimated cost-effectiveness for 20 different groups. Women entered the model at the age of 25 years, and exited the model either on death or when they reached 70 years of age, when routine surveillance ends and any further surveillance beyond this age needs to be agreed by the woman’s general practitioner.

Participants were characterised by their CRA-based and PRA-based risk groups. They then transitioned between six health states: healthy, BC, BC survivor (BC survivors), OC, OC survivor (OC survivors) and dead. In the BC and OC states, 10 separate categories were used to include patients who were 1–10 years postdiagnosis. They then enter the ‘survivor’ status at 10 years after diagnosis, if they survived until then.

Comparisons were made between two scenarios:

1. CRA: individuals were classified into high-risk, moderate-risk or near-population-risk groups for BC and OC based on their GPV status and family history. Details for risk classification are provided in online supplemental material 1.
2. PRA: following genetic testing, participants were given a personalised risk estimate. Their risk scores were calculated using the CanRisk cancer risk prediction tool, which combines the patient’s genetic result, family history and PRS, along with hormonal and lifestyle factors.^24^ Three models were used, which were the combinations of QRF and PRS. MD was not used.

In both scenarios, after individuals were classified into high-risk, moderate-risk and near-population-risk categories for BC and OC, they were assumed to receive clinical management according to their risk category based on the National Institute for Health and Care Excellence (NICE) guidelines and published literature.^25^,^27^ For BC, those at high risk were given the option of RRM and risk-reducing medication, plus annual mammography from ages 40 to 59 years, with triennial screening (as per NHS Breast Screening Programme) from age 60 to 69 years. MRI screening was offered annually for those with GPVs in *BRCA1*/*BRCA2*/*PALB2* from age 30 to 49 years and classified as high risk, and they were offered annual mammograms from age 40 to 69 years. Those at moderate risk were given annual mammography from ages 40 to 49 years, and triennial screening from 50 to 69 years. Those in the near-population-risk category were given triennial mammographic screening from ages 50 to 69 years.^25^ For OC, those at high risk were given the option of RRSO. None of the risk groups for OC were offered surveillance, as there is currently no effective surveillance programme for this cancer^26^ (table 1).

**Table 1:** Clinical management by risk category^11 25 26^

BC lifetime risk category		BC	OC lifetime risk category		OC
<17%	Near-population	Mammographic screening: triennial, age 50–69 years	<5%	Near-population	No surveillance
≥17% to <30%	Moderate	Mammographic screening: annual, age 40–49 years; triennial, age 50–69 years.			
≥30%	High	Mammographic screening: annual, age 40–59 years; triennial, age 60–69 years. Offered risk-reducing mastectomy. Offered risk-reducing medication.	≥5%	High	No surveillance; offered risk-reducing salpingo-oophorectomy
*BRCA1*/*BRCA2*/*PALB2* mutation carriers (high-risk category)		MRI screening: annual, age 30–49 years Mammographic screening: annual, age 40–69 years Offered risk-reducing mastectomy Offered risk-reducing medication			

BC, breast cancer; OC, ovarian cancer.

### Risk distribution matrices

The distribution of individuals by their BC and OC risk (the underlying cancer incidence rate used in the simulation model) and estimated BC and OC risk (the risk used to decide the clinical management for each patient) were presented in a 9×9 matrix. The actual risk came from a risk category distribution generated by the most comprehensive version of PRA (PRS and QRF).^10^ Benchmark BC and OC incidences for risk categories were calibrated to this ‘gold standard’ as described in online supplemental material 3. All participants were assumed to have true cancer risks as calculated via this method. Estimated risk distributions were calculated for CRA and the three versions of PRA according to the data shown in online supplemental material 1.^13^

### Parameters for simulation

**Figure 1:**
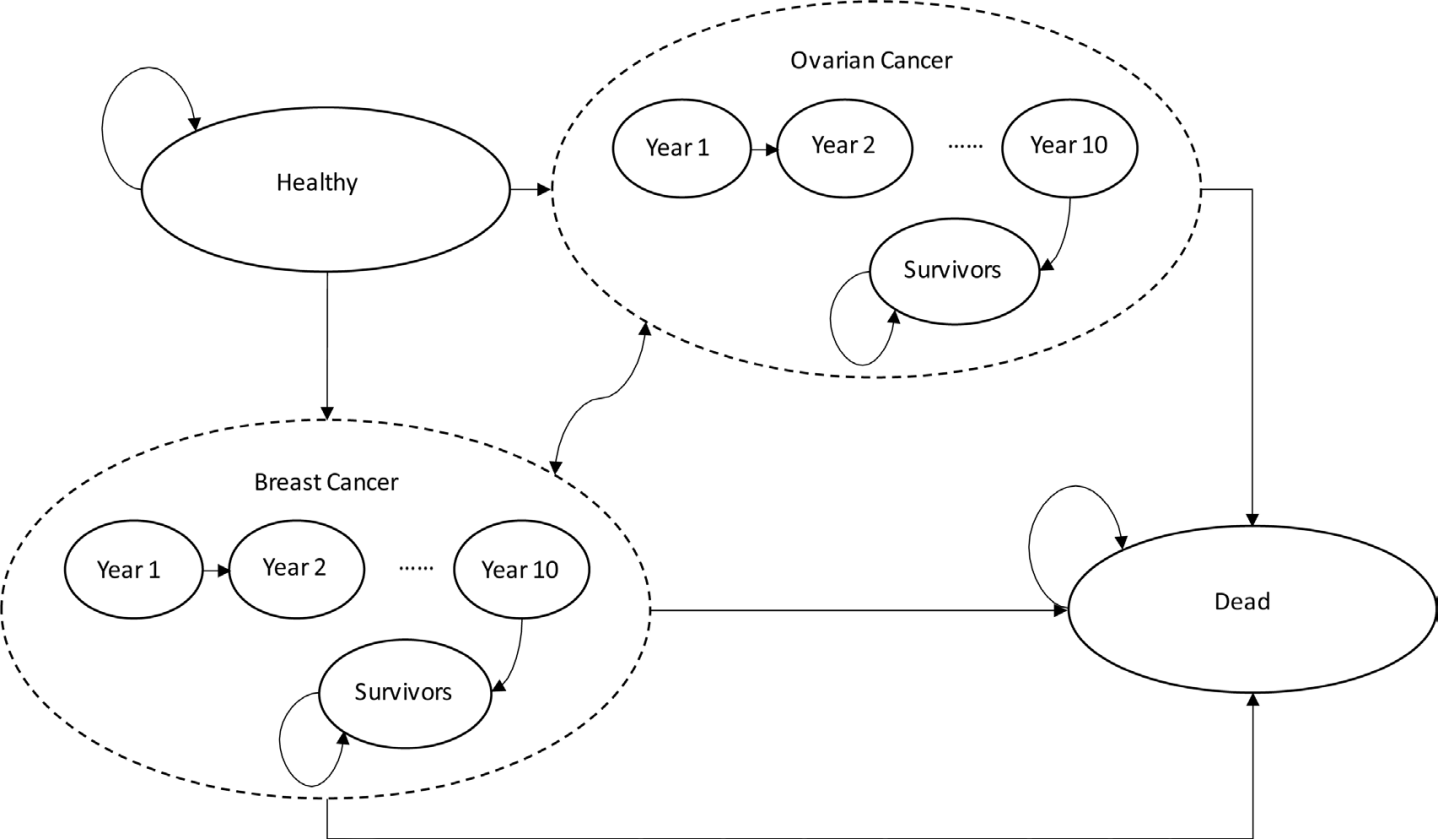
Patient-level Markov simulation.

The simulation model is shown in figure 1 and parameters for the simulation are provided in table 2. The baseline incidence rate of BC and OC for all women in the UK by age were drawn from Cancer Research UK.^28^ Relative risks of BC and OC incidence for each risk group (near-population, moderate, high) compared with the incidence rate for the general population were multiplied by a factor.^6 29^ The relative risk was calibrated to the most comprehensive estimation from the PRA, which included QRF and PRS. All calibrations were conducted in the software package R.^30^ Fittings were performed with the Bhat package based on the least squares method by minimising the sum of residuals.^31^ See online supplemental material 3 for details of the model calibration strategy.

**Table 2:** Parameters for the simulation model

Parameter name	Data
All-cause mortality, by age	0–0.35^32^
Incidence rate of breast cancer, by age	0–0.004^28^
Incidence rate of ovarian cancer, by age	0–0.0007^28^
1-Year/5-Year/10-Year survival rate of breast cancer	0.958/0.85/0.759^28^
1-Year/5-Year/10-Year survival rate of ovarian cancer	0.717/0.426/0.353^28^
Uptake rate of risk-reducing medication	0.163^17^
HR of breast cancer incidence under risk-reducing medication	0.71^17^
Uptake rate of risk-reducing mastectomy	0.47^17^
Risk reduction from risk-reducing mastectomy	0.91^17^
Uptake rate of risk-reducing salpingo-oophorectomy	0.55^17^
Risk reduction from risk-reducing salpingo-oophorectomy	0.95^17^
Relative mortality of breast cancer from screening	0.8^20^

The all-cause mortality rates for all women in the UK by age were drawn from the Office for National Statistics.^32^ The death rate for patients with BC and OC by time since diagnosis was calculated based on the 1-year/5-year/10-year survival rates of BC drawn from Cancer Research UK.^28^ Individuals entered the survivor category after 10 years from diagnosis, and followed the population all-cause mortality for their respective ages. When the BC or OC death rate was lower than the population all-cause mortality, the latter was used instead of the cancer-specific death rate. The uptake rate and risk reduction rate of RRM and RRSO were based on published literature.^17^ Regular mammographic screening was assumed to have no influence on BC incidence, but could reduce mortality.^20^

The relative prevalence of GPVs was drawn from CanRisk estimations.^24^ Details are in online supplemental material 2.

### Measuring costs

Cost data are provided in table 3 and include genetic testing cost, cost of PRA, cost of risk-reducing surgery/medication, cost of surveillance and treatment cost in each health state (diagnosis and surgery for cancer, radiotherapy, chemotherapy, medications, terminal care). Unit costs were derived from published literature,^33^ NHS reference costs^34^ and the British National Formulary.^35^ Costs prior to 2023 were transformed to 2023 £ prices based on the NHS Gross Domestic Product deflator.^36^

**Table 3:** Costs and health utilities

Cost (£)	
Conventional risk assessment	240^11 36^
Personalised risk assessment	357–388 (see online supplemental material 3)
Risk-reducing medication	159^36 38^
Risk-reducing mastectomy	4836^11 36^
Risk-reducing salpingo-oophorectomy	4253^11 36^
Breast cancer diagnosis and treatment, year 1	21 319^11 36^
Breast cancer diagnosis and treatment, years 2–5	2407^11 36^
Ovarian cancer diagnosis and treatment, year 1	23 273^11 36^
Ovarian cancer diagnosis and treatment, year 2	6406^11 36^
Ovarian cancer diagnosis and treatment, years 3–5	5967^11 36^
Breast/Ovarian cancer terminal care	18 514^11 36^
Breast cancer screening programme, population	416^11 36^
Breast cancer screening programme, *BRCA1/BRCA2/PALB2*	5491^11 36^
Breast cancer cost for triennially screened versus unscreened	−10%^20^
Breast cancer cost for annually versus triennially screened	−6%^37^
Utility scores	
Aged-based utility: 35–44, 45–54, 55–64, 65–74, 75+ years	0.91, 0.85, 0.81, 0.78, 0.71^33^
Breast cancer, years 1–5	0.71, 0.72, 0.73, 0.74, 0.76^33^
Breast cancer, years 6+	0.77^33^
Ovarian cancer, years 1–5	0.5, 0.65, 0.67, 0.69, 0.7^33^
Ovarian cancer, years 6+	0.72^33^
Risk-reducing mastectomy	0.88^38^
Risk-reducing salpingo-oophorectomy	0.95^38^
Relative breast cancer utility of unscreened versus screened	0.94^21^

Populations under different screening strategies were assigned different estimated costs, which were calculated by applying multipliers to the benchmark cost of a population screening programme to calculate the screening cost for those undergoing intensified screening due to their higher risks.^11 36^ The multipliers were based on the total times of screening occurring in the lifetime of an individual in different risk categories.

For diagnosis and treatment of BC and OC, we included diagnostic costs in the clinic, treatment costs and end-of-life costs. Treatment costs were applied according to the time since diagnosis for years 1–5 with an additional cost associated with end of life applied to the year before death, where this occurred. For those under annual breast screening or no screening, a multiplier was applied to the screening cost to differentiate them from those under the standard triennial screening, since they were likely to be at different stages of cancer.^20 37^ The end-of-life cost was only applied to patients with cancer who died, but not individuals who died from other causes.

### Measuring health outcomes

Health utility values are provided in table 3. Healthy individuals were given the age-based utility score for UK women, while those who died were assigned a utility score of 0.^33^ For other health states, the age-based score for healthy individuals was multiplied by a health state-based utility value.^33^ The values of the health state-based utility values were drawn from published literature. Screened and unscreened populations were given different health utility values on diagnosis of BC, since they were likely to be diagnosed at different stages.^21^ Since RRM and RRSO may lead to side effects, a 1-year disutility was applied to those undertaking these surgeries.^38^

### Measuring cost-effectiveness

The overall cost and utility for each scenario were calculated as weighted averages of the costs and utilities for patients in each risk category, with the weights based on the proportion of patients in each category. A deterministic sensitivity analysis was performed by increasing the cost of performing risk assessment by 30%. A probabilistic sensitivity analysis was performed simultaneously comparing all four options jointly (CRA, QRF, PRS, QRF and PRS) in each of the 20 groups. To do this, we applied a multinomial distribution to the 9×9 risk distribution matrix and a gamma distribution to the additional cost of PRA. We ran 1000 replications of the model for each of the 20 groups, allowing the parameters to vary and calculated the net monetary benefit (QALY×willingness to pay (WTP) threshold for a QALY−cost) of each option across a range of values for the maximum WTP for a QALY (from £0 to £40 000). We computed cost-effectiveness acceptability curves showing the probability that the four options were cost-effective based on the proportion of the replications where each option had the highest net monetary benefit at each WTP threshold value.

## Results

### Cost-effectiveness

The analysis was performed for 20 groups based on GPV status and family history. See online supplemental table for detailed results.

Among women with GPV in genes that were high-risk for BC and OC (group HH*—BRCA1*, *BRCA2*), CRA was the most cost-effective in all family history scenarios.

Among women with GPV in genes that were high-risk for BC and moderate-risk for OC (group HN*—PALB2*), CRA was the most cost-effective among those with unknown family history or BC family history. PRS and QRF were the most cost-effective for those with OC family history, and PRS was the most cost-effective for those with BC+OC family history.

Among women with GPV in genes that were moderate-risk for BC and high-risk for OC (group MH*—RAD51C*, *RAD51D*), PRS was the most cost-effective among those with BC, OC or BC+OC family history, although in the BC and BC+OC family history groups, CRA generated the highest QALYs. QRF and PRS was the most cost-effective for those with unknown family history.

Among women with GPV in genes that were moderate-risk for BC and OC (group MN*—ATM*, *CHEK2*), PRS was the most cost-effective for women with all family histories.

Among women with no pathogenic variant, CRA was the most cost-effective in all family history scenarios, although PRS and QRF generated the highest QALYs in unknown family history and OC family history groups.

### Sensitivity analysis

We performed a univariate deterministic sensitivity analysis by increasing the cost of risk assessment by 30% (online supplemental table S3).

**Figure 2:**
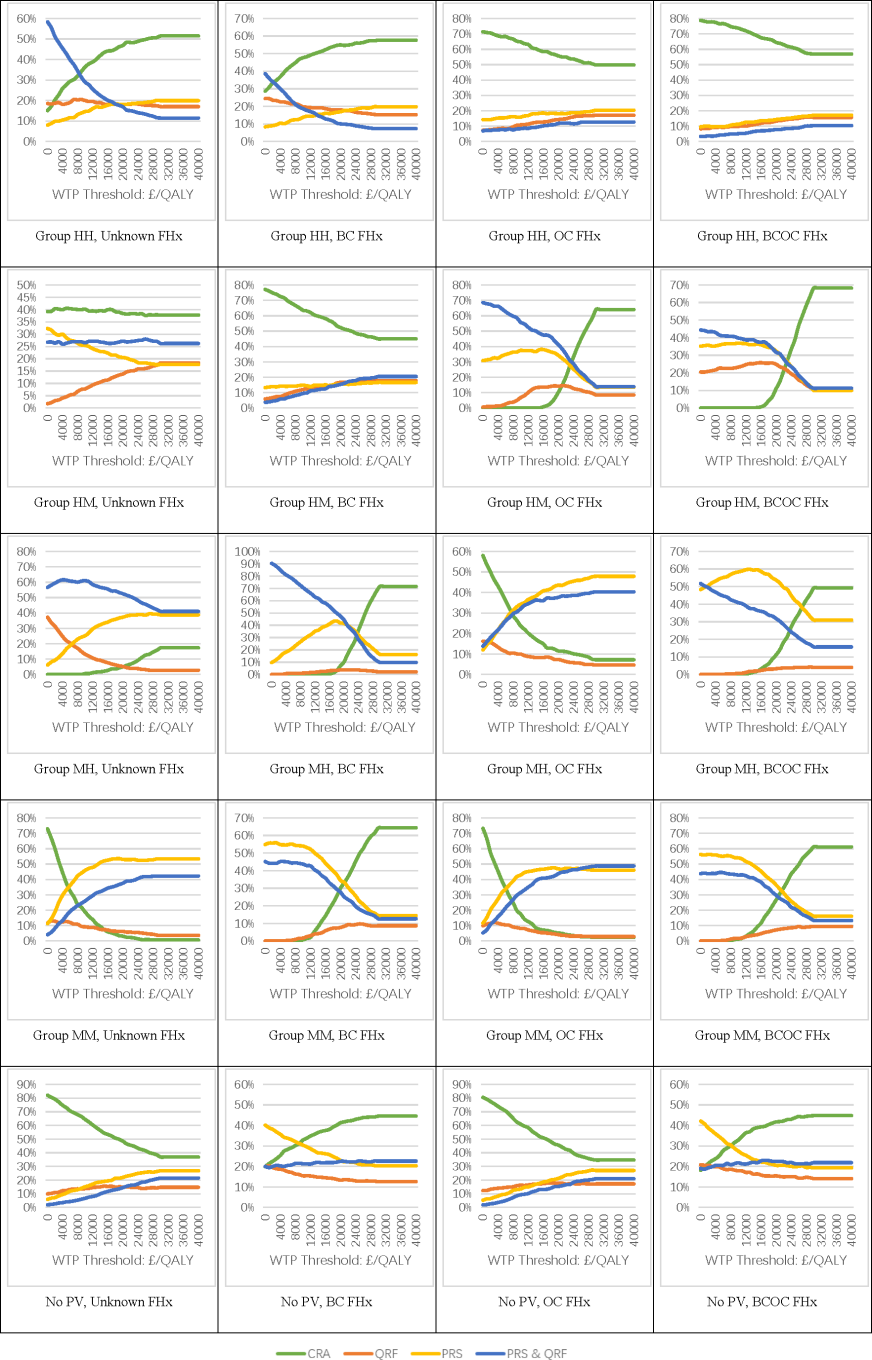
Probability of each strategy being the most cost-effective option. BC, breast cancer; FHx, family history; OC, ovarian cancer; QALY, quality-adjusted life year; WTP, willingness to pay.

Figure 2 shows the results of the probabilistic sensitivity analysis. The x-axis represents the different WTP thresholds for a QALY, from £0 to £40 000/QALY, while the y-axis represents the probability that a risk stratification method is the most cost-effective option among all methods—CRA, PRA via QRS, PRA via PRS and PRA via QRS and PRS. The conclusions were similar to those in the base case analysis (figure 2).

## Discussion

The economic evaluation showed that PRA was cost-effective compared with CRA in certain specific situations based on only HBOC genetic testing. These findings remained stable under sensitivity analysis.

The results showed that PRA could be cost-effective for some groups of women under the NICE-recommended WTP threshold with current cost and utility data, and provide evidence for policy making. In particular, PRA is likely to be cost-effective in groups where it is likely to produce a different (more accurate) classification of BC/OC risk than CRA. In groups where the risk classification is not different between PRA and CRA, CRA would generally be more cost-effective due to its lower cost. They show the practical value of introducing PRA components like PRS and QRF information into clinical practice to supplement or replace the current risk assessment based on genetic test results only. Implementation of PRA could improve accuracy in providing more effective risk stratification for BC and OC at an acceptable cost, in turn leading to more efficient allocation of resources for the prevention and surveillance of BC and OC.

The findings also revealed the need to collect more relevant risk factor information in order to achieve optimal risk stratification for HBOC. PRS could be generated as part of the HBOC predictive test, and the use of the patient-facing risk factor collection tools will facilitate the routine collection of QRF data.

This study was the first economic evaluation to compare PRA with CRA for women with or without GPVs in BC and OC susceptibility genes. The results indicated the economic feasibility of introducing PRA into the BC and OC risk stratification for women. A previous study had shown that BC and OC prevention options were cost-effective for GPV carriers and further showed that prevention strategies based on more accurate risk stratification would be necessary.^19^

There were several limitations. First, this was a hypothetical model. The risk category distribution of GPV carriers was based on mathematical calculation rather than results from an actual clinical trial. The cancer incidence rates were calibrated to the distribution generated via estimation. This could lead to uncertainties in downstream economic evaluations. Second, the cost for PRA was based on estimates from a single NHS Clinical Genetics service. These estimates were permissive and may affect the accuracy of the incremental cost-effectiveness ratio (ICER) values. Third, we did not include risk estimation methods incorporating MD information due to the limited availability of such data, which is typically only available at later ages and may change over time. Fourth, we did not consider more risk-adapted screening approaches, where individuals at very low risk may be offered less frequent screening. Lastly, the analyses do not consider more complex scenarios that may occur in practice, such as PRA informing decisions on the timing of different interventions.

## Conclusion

PRA for hereditary BC and OC was cost-effective in certain situations compared with CRA for women with or without GPVs in HBOC genes in the UK NHS system.

## Supplementary material

10.1136/jmg-2024-109948 online supplemental file 1

10.1136/jmg-2024-109948 online supplemental file 2

## Data Availability

Data are available on reasonable request.
