# Supplementary material for: Economic evaluation of personalised versus conventional risk assessment for women who have undergone testing for hereditary breast and ovarian cancer genes: a modelling study
Source: J Med Genet. 2025 Apr 10;62(7):e109948. doi: 10.1136/jmg-2024-109948 (PMC12322399; doi:10.1136/jmg-2024-109948)
Supplement: online supplemental file 1 [file jmg-62-7-s001.docx]

Supplementary Material 1. Population cancer risk distribution under conventional and personalized risk assessment^1,2^

Table S1.1 Distribution of population among breast cancer risk groups (unknown family history)

|  |  |  | **Conventional Risk Assessment** | | | | | | | |
| --- | --- | --- | --- | --- | --- | --- | --- | --- | --- | --- |
|  | | *Mutation* | *BRCA1* | *BRCA2* | *PALB2* | *CHEK2* | *ATM* | *RAD51C* | *RAD51D* | *No PV* |
|  |  | Risk | 75.4 | 76.9 | 52.4 | 22.9 | 23.6 | 22.3 | 20.8 | 11.6 |
|  |  | Risk Category | *High* | | | *Moderate* | | | | *Near-Population* |
| Personalized Risk Assessment | *QRF* | *Near-Population* | 0.0 | 0.0 | 0.0 | 5.3 | 4.1 | 9.3 | 16.6 | 97.0 |
|  |  | *Moderate* | 0.0 | 0.0 | 0.0 | 89.4 | 87.7 | 86.1 | 81.3 | 3.0 |
|  |  | *High* | 100.0 | 100.0 | 100.0 | 5.3 | 8.2 | 4.5 | 2.1 | 0.0 |
|  | *PRS* | *Near-Population* | 0.0 | 0.0 | 0.0 | 26.9 | 23.1 | 28.2 | 35.1 | 86.8 |
|  |  | *Moderate* | 0.0 | 1.5 | 3.2 | 53.6 | 56.1 | 55.1 | 52.5 | 12.9 |
|  |  | *High* | 100.0 | 98.5 | 96.8 | 19.5 | 20.9 | 16.7 | 12.4 | 0.4 |
|  | *QRF & PRS* | *Near-Population* | 0.0 | 0.0 | 0.1 | 30.1 | 26.8 | 31.6 | 38.0 | 84.9 |
|  |  | *Moderate* | 0.0 | 2.5 | 5.2 | 48.7 | 50.5 | 49.5 | 47.4 | 14.4 |
|  |  | *High* | 100.0 | 97.4 | 94.8 | 21.2 | 22.7 | 18.8 | 14.6 | 0.8 |

Table S1.2 Distribution of population among breast cancer risk groups (mother affected at age 50)

|  |  |  | **Conventional Risk Assessment** | | | | | | | |
| --- | --- | --- | --- | --- | --- | --- | --- | --- | --- | --- |
|  | | *Mutation* | *BRCA1* | *BRCA2* | *PALB2* | *CHEK2* | *ATM* | *RAD51C* | *RAD51D* | *No PV* |
|  |  | Risk | 80.0 | 67.3 | 62.9 | 33.3 | 34.1 | 32.5 | 30.6 | 18.0 |
|  |  | Risk Category | *High* | | | | | | | *Moderate* |
| Personalized Risk Assessment | *QRF* | *Near-Population* | 0.0 | 0.0 | 0.0 | 0.0 | 0.0 | 0.0 | 0.0 | 41.4 |
|  |  | *Moderate* | 0.0 | 0.0 | 0.0 | 26.4 | 24.1 | 32.3 | 45.8 | 58.3 |
|  |  | *High* | 100.0 | 100.0 | 100.0 | 73.7 | 75.9 | 67.7 | 54.2 | 0.3 |
|  | *PRS* | *Near-Population* | 0.0 | 0.0 | 0.0 | 5.9 | 4.2 | 5.9 | 8.6 | 59.7 |
|  |  | *Moderate* | 0.0 | 0.2 | 0.4 | 45.0 | 43.4 | 48.1 | 53.0 | 37.7 |
|  |  | *High* | 100.0 | 99.8 | 99.6 | 49.1 | 52.4 | 46.1 | 38.4 | 2.7 |
|  | *QRF & PRS* | *Near-Population* | 0.0 | 0.0 | 0.0 | 8.8 | 7.1 | 9.2 | 12.4 | 60.1 |
|  |  | *Moderate* | 0.0 | 0.5 | 1.0 | 42.9 | 41.8 | 45.3 | 48.8 | 35.5 |
|  |  | *High* | 100.0 | 99.6 | 99.0 | 48.2 | 51.1 | 45.5 | 38.8 | 4.4 |

Table S1.3 Distribution of population among ovarian cancer risk groups (unknown family history)

|  | | | **Conventional Risk Assessment** | | | | | |
| --- | --- | --- | --- | --- | --- | --- | --- | --- |
|  | | *Mutation* | *BRCA1* | *BRCA2* | *RAD51D* | *RAD51C* | *PALB2* | *No PV* |
|  |  | Risk | 44.3 | 14.7 | 12.8 | 11.0 | 5.0 | 1.7 |
|  |  | Risk Category | *High* | | | | *Moderate* | *Near-Population* |
| Personalized Risk Assessment | *RF* | *Near-Population* | 0.0 | 0.1 | 0.5 | 1.8 | 41.2 | 56.9 |
|  |  | *Moderate* | 0.0 | 12.7 | 24.4 | 44.0 | 54.4 | 41.6 |
|  |  | *High* | 100.0 | 87.2 | 75.1 | 54.2 | 4.4 | 1.4 |
|  | *PRS* | *Near-Population* | 0.0 | 0.0 | 0.0 | 0.2 | 31.7 | 53.9 |
|  |  | *Moderate* | 0.0 | 6.1 | 17.5 | 38.6 | 67.2 | 45.9 |
|  |  | *High* | 100.0 | 93.9 | 82.5 | 61.3 | 1.1 | 0.2 |
|  | *RF & PRS* | *Near-Population* | 0.0 | 0.8 | 2.0 | 4.8 | 44.2 | 57.8 |
|  |  | *Moderate* | 0.0 | 19.6 | 30.3 | 42.8 | 48.5 | 38.6 |
|  |  | *High* | 100.0 | 79.6 | 67.7 | 52.4 | 7.3 | 3.6 |

Table S1.4 Distribution of population among ovarian cancer risk groups (mother affected at 50)

|  | | | **Conventional Risk Assessment** | | | | | |
| --- | --- | --- | --- | --- | --- | --- | --- | --- |
|  | | *Mutation* | *BRCA1* | *BRCA2* | *RAD51D* | *RAD51C* | *PALB2* | *No PV* |
|  |  | Risk | 58.8 | 26.1 | 23.2 | 20.1 | 10.1 | 3.5 |
|  |  | Risk Category | *High* | | | | | *Near-Population* |
| Personalized Risk Assessment | *RF* | *Near-Population* | 0.0 | 0.0 | 0.0 | 0.0 | 3.3 | 87.8 |
|  |  | *Moderate* | 0.0 | 0.2 | 0.6 | 2.0 | 52.3 | 12.1 |
|  |  | *High* | 100.0 | 99.9 | 99.4 | 98.0 | 44.4 | 0.1 |
|  | *PRS* | *Near-Population* | 0.0 | 0.0 | 0.0 | 0.0 | 0.2 | 97.6 |
|  |  | *Moderate* | 0.0 | 0.0 | 0.0 | 0.1 | 57.1 | 2.4 |
|  |  | *High* | 100.0 | 100.0 | 100.0 | 99.9 | 42.7 | 0.0 |
|  | *RF & PRS* | *Near-Population* | 0.0 | 0.0 | 0.0 | 0.1 | 7.2 | 86.7 |
|  |  | *Moderate* | 0.0 | 0.8 | 1.9 | 4.9 | 52.0 | 13.1 |
|  |  | *High* | 100.0 | 99.2 | 98.1 | 95.1 | 40.8 | 0.2 |

- *Note: The risk category distributions were based on the implied risk distributions in the models described in Lee et al. paper^2^*

Supplementary Material 2. Relative prevalence of pathogenic variants^3^

Female #1: born in 1999, unknown family history


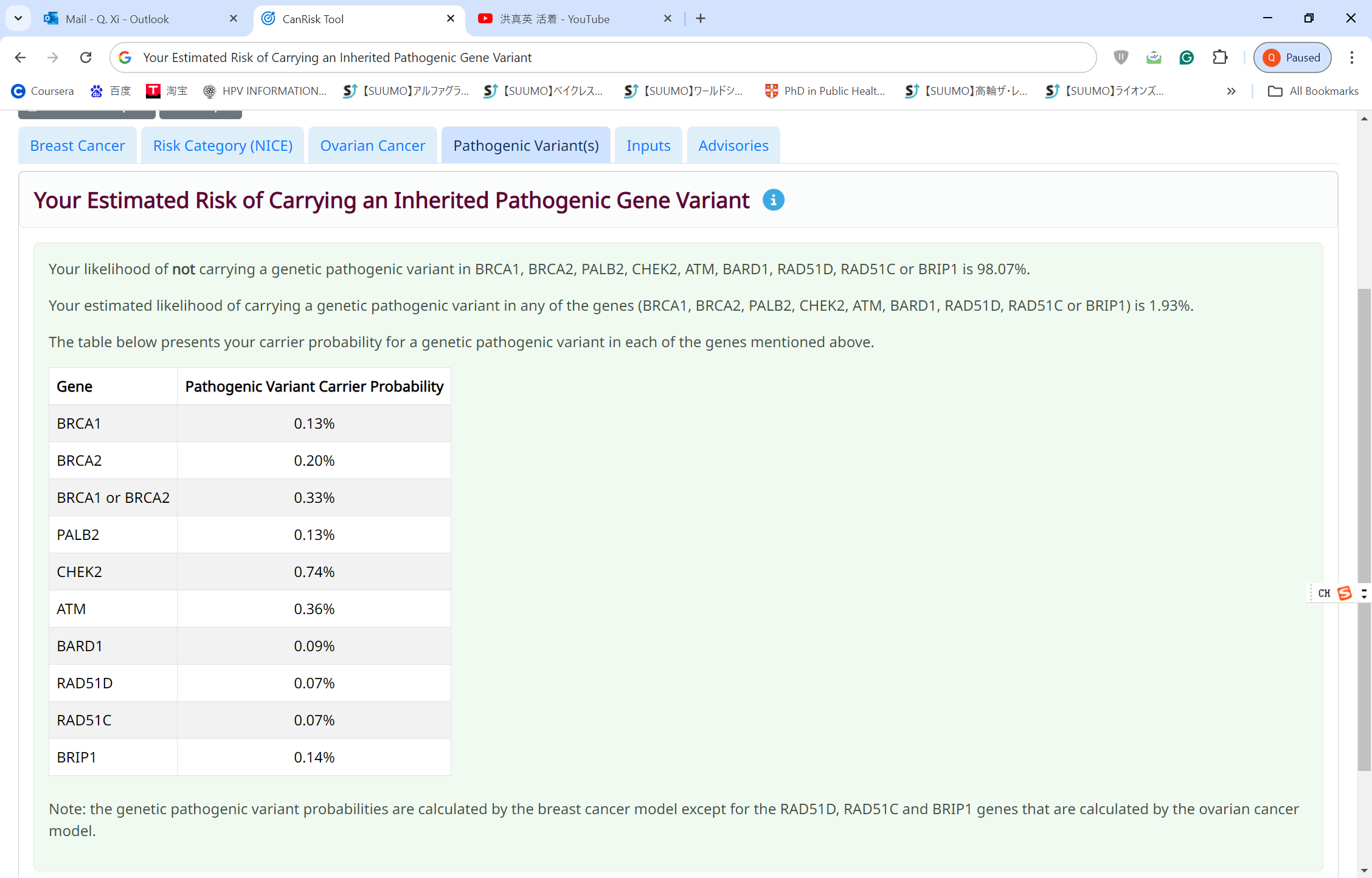


Female #2: born in 1999, mother affected by BC at 50


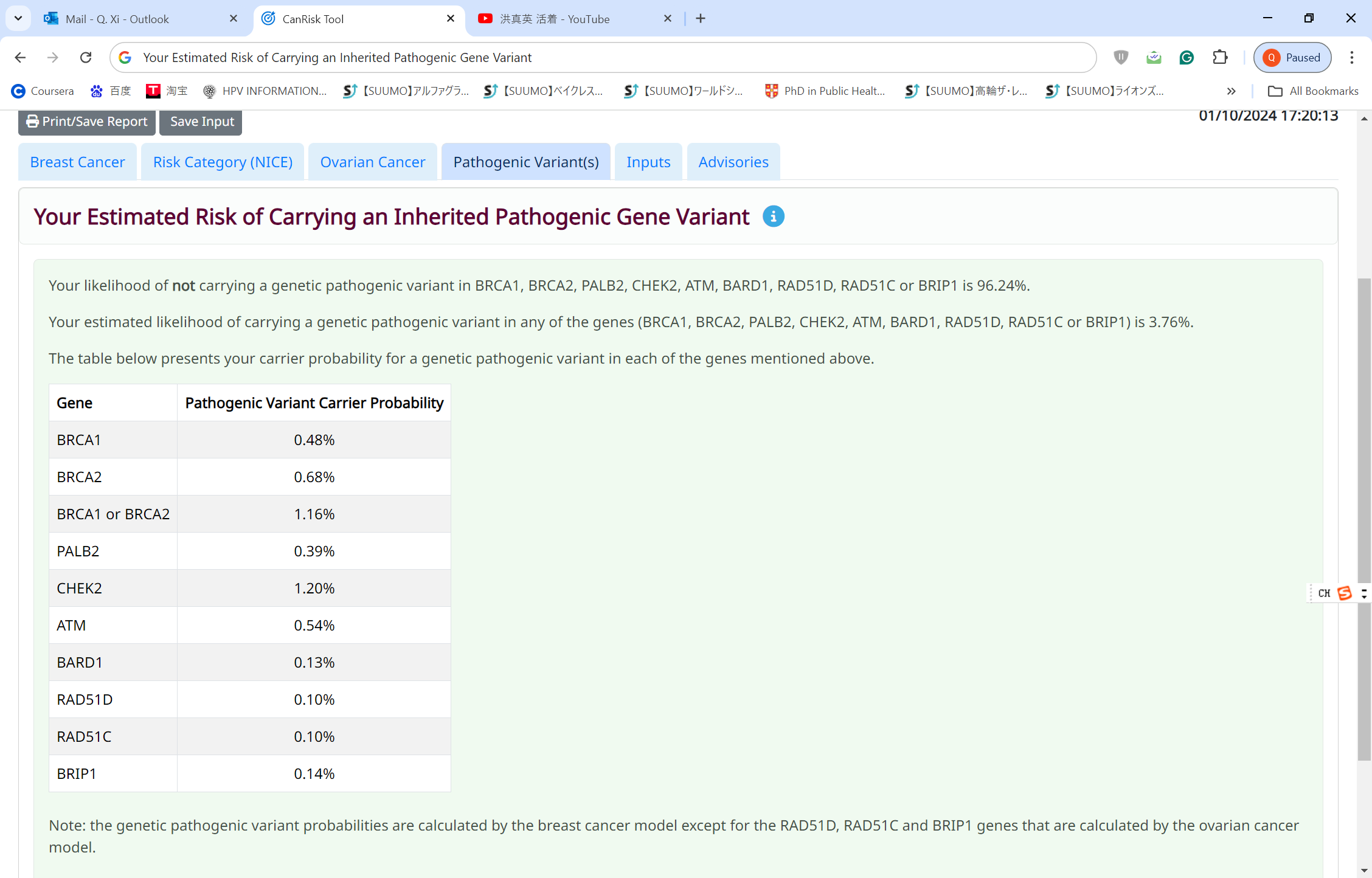


Female #3: born in 1999, mother affected by OC at 50


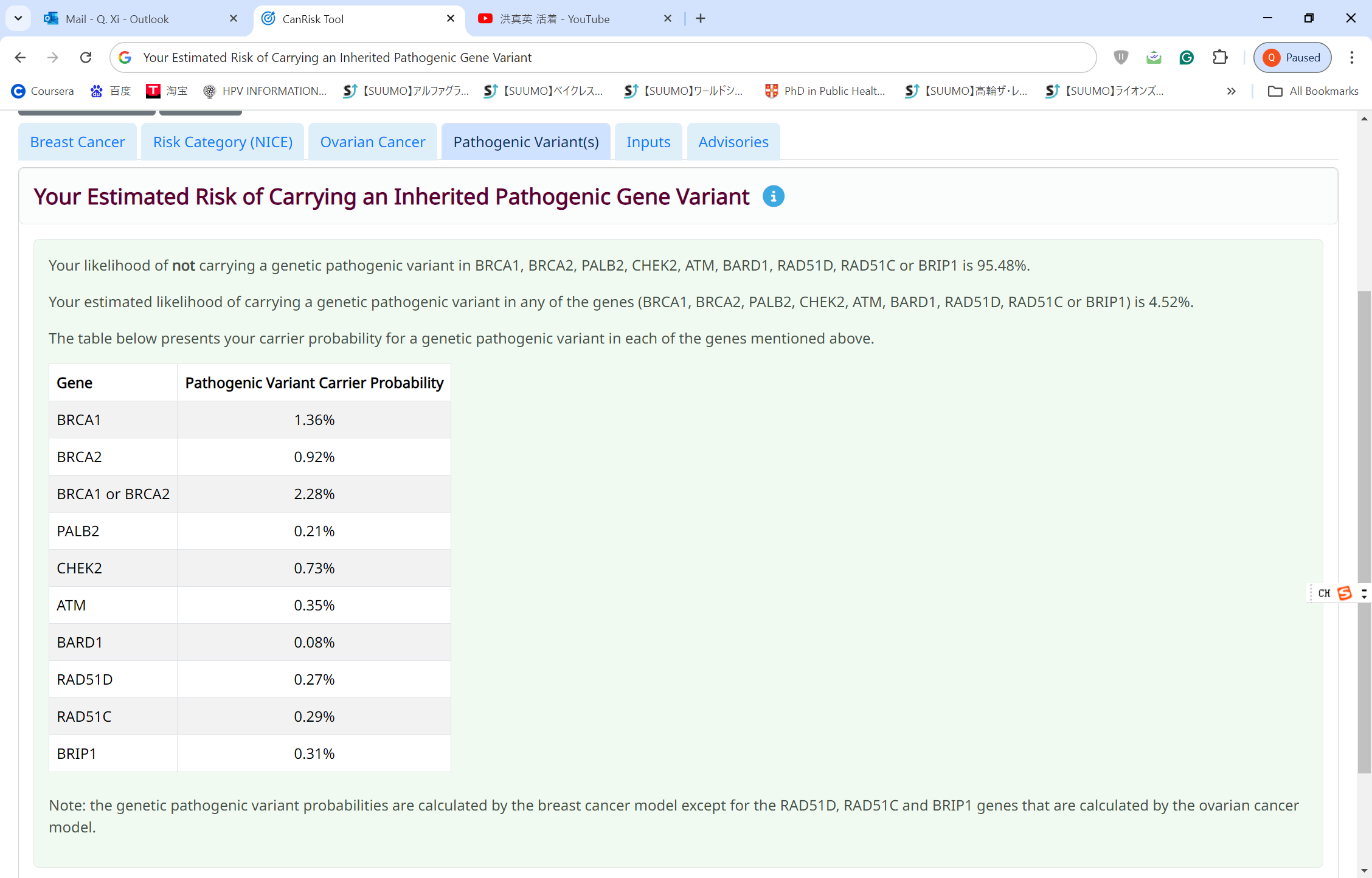


Female #4: born in 1999, mother affected by BC and OC at 50


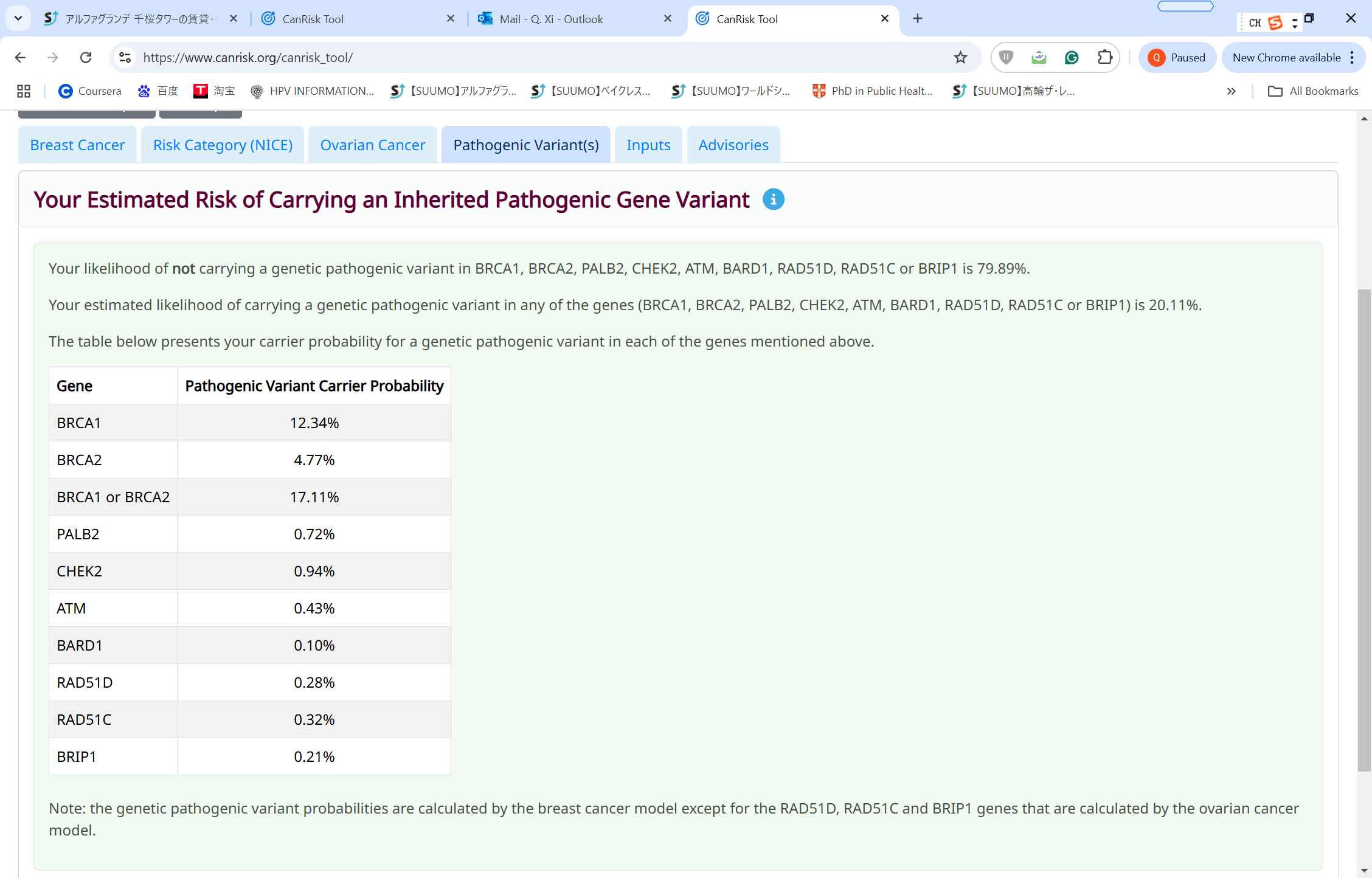


Supplementary Material 3. Relative risk of cancer incidence for each risk category

1. *Calculating median lifetime risk for each risk category*

A log-normal distribution was fitted to the included population according to the distribution of risk groups. The expected lifetime risk of each group could be calculated from the fitted distribution. Specifically, $\ln\left( Risk \right)\sim N\left( \mu, \sigma^{2} \right)$ in which N was a normal distribution with mean $\mu$ and standard deviation $\sigma$. Parameters $\mu$ and $\sigma$ for the distribution were calibrated to two known points: $ln\left( {Risk}_{pop,mod} \right)=Qnorm\left( {Prop}_{pop}, \mu,\sigma^{2} \right);$ $ln\left( {Risk}_{mod, high} \right)=Qnorm({Prop}_{pop}+{Prop}_{mod}, \mu, \sigma^{2})$. ${Risk}_{pop,mod}$ referred to the split point for population and moderate risk, which is 17%; and ${Risk}_{mod,high}$ referred to the split point for moderate and high risk, which is 30%. The estimation of the distribution $N(\mu, \sigma^{2})$ was conducted using the least squares method by minimizing the sum of residuals: ${(({Risk}_{pop,mod}| \mu,\sigma^{2})-{Risk}_{pop,mod})}^{2}+{{((Risk}_{mod,high}| \mu,\sigma^{2})-{Risk}_{mod,high})}^{2}$.

With the fitted risk distribution, the expected lifetime risk for each risk group could be estimated. Specifically, the expected cancer lifetime risk for near-population, moderate, and high risk groups were: ${Risk}_{pop}=e^{Qnorm({Prop}_{pop}/2, \mu,\sigma^{2})}; {Risk}_{mod}=e^{Qnorm({Prop}_{pop}+{Prop}_{mod}/2, \mu,\sigma^{2})}; {Risk}_{high}=e^{Qnorm({Prop}_{pop}+{Prop}_{mod}+{Prop}_{high}/2, \mu,\sigma^{2})}$.

1. *Calculating relative risk of incidence for each risk category*

The relative risk for each risk group ($\lambda_{r})$was multiplied onto population age-based cancer risk incidence data into the Markov simulation, and simulation results of lifetime cancer incidence were fitted to the expected lifetime risk for their respective risk categories. The lifetime incidence rate of cancer estimated by implementing $\lambda_{pop}, \lambda_{mod}, \lambda_{high}$ into the simulation model was compared to the ${Risk}_{pop}, {Risk}_{mod}, {Risk}_{high}$ calculated above. The estimation of $\lambda_{r}$ was performed according to least square method by minimizing the residuals: ${({(Risk}_{r}|\lambda_{r})-{Risk}_{r})}^{2}$.

Supplementary Material 4. Cost of conventional and personalized risk assessment

- *Figure* S*1. Flowchart for processes in conventional and personalized risk assessment*


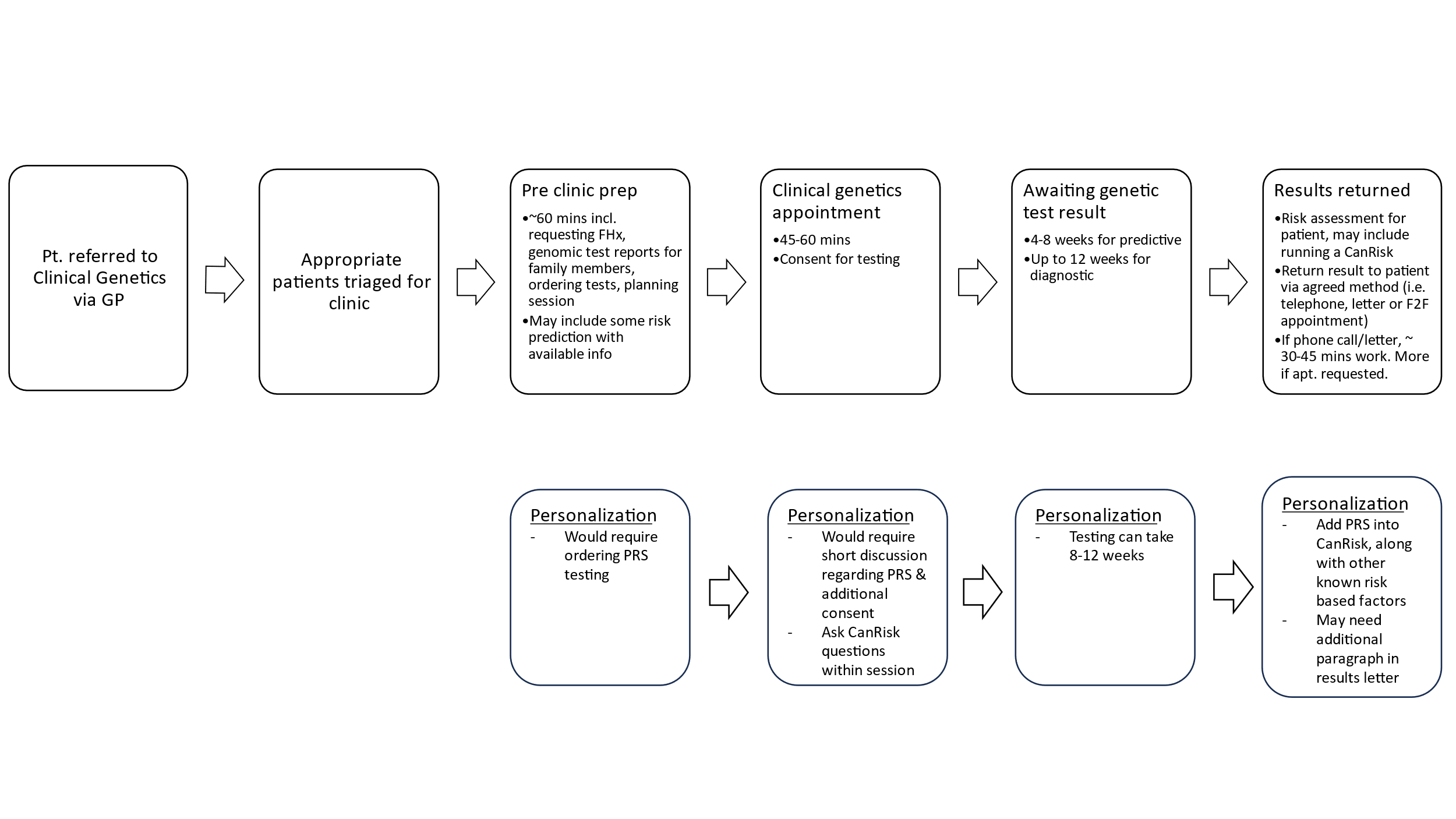


- *Note: The upper half are the stages for conventional risk assessment. The lower half are the additional tasks in each stage for personalized risk assessment.*

Table S2. Tasks associated with risk assessment methods and costs

| *Task* | *Method* | *Staff* | *Band* | *Time (min)* | *Pay per hour (UK£)^4^* | *Cost (UK£)* |
| --- | --- | --- | --- | --- | --- | --- |
| *Conventional Risk Assessment* | | | | | | |
|  | 240^5,6^ | | | | | |
| *Additional Cost for Personalized Risk Assessment* | | | | | | |
| Risk assessment | *PRS* | Scientist | 6/7 | 30 | 60.5 | 30.25 |
|  | *QRF* | Genetic counsellor | 7 | 30 | 66 | 33 |
| Initial data collection & Results appointment | | Genetic counsellor | 7 | 60 | 66 | 66 |
|  |  | Administrator | 4 | 30 | 37 | 18.5 |

Supplementary Material 5. Deterministic sensitivity analysis

Table S3. Increasing risk assessment cost by up to 30%

|  | *Cost (UK£)* | | | | *QALY* | | *ICER* | | |
| --- | --- | --- | --- | --- | --- | --- | --- | --- | --- |
| Method* | *Cancer* | *Risk* | *Total* | *Δ* |  | *Δ* | *Full Incremental Analysis* | | |
| *Group HH, Unknown FHx* | | | | | | | | | |
| CRA | 8676 | 312 | 8988 |  | 19.097 |  |  |  |  |
| QRF&PRS | 8527 | 461 | 8988 | 1 | 19.091 | -0.006 | Dominated |  |  |
| QRF | 8603 | 422 | 9025 | 37 | 19.094 | -0.004 | Dominated |  |  |
| PRS | 8622 | 418 | 9040 | 52 | 19.095 | -0.002 | Dominated |  |  |
| *Group HH, BC FHx* | | | | | | | | | |
| CRA | 9085 | 312 | 9397 |  | 19.024 |  |  |  |  |
| QRF&PRS | 8967 | 461 | 9428 | 31 | 19.019 | -0.005 | Dominated |  |  |
| QRF | 9015 | 422 | 9436 | 40 | 19.021 | -0.003 | Dominated |  |  |
| PRS | 9049 | 418 | 9467 | 70 | 19.022 | -0.002 | Dominated |  |  |
| *Group HH, OC FHx* | | | | | | | | | |
| CRA | 10302 | 312 | 10614 |  | 18.601 |  |  |  |  |
| PRS | 10290 | 418 | 10708 | 94 | 18.601 | 0.000 | Dominated |  |  |
| QRF | 10302 | 422 | 10724 | 109 | 18.601 | 0.000 | Dominated |  |  |
| QRF&PRS | 10278 | 461 | 10739 | 125 | 18.600 | -0.001 | Dominated |  |  |
| *Group HH, BCOC FHx* | | | | | | | | | |
| CRA | 11979 | 312 | 12291 |  | 18.139 |  |  |  |  |
| PRS | 11978 | 418 | 12396 | 105 | 18.138 | 0.000 | Dominated |  |  |
| QRF | 11979 | 422 | 12401 | 109 | 18.138 | 0.000 | Dominated |  |  |
| QRF&PRS | 11975 | 461 | 12436 | 145 | 18.138 | 0.000 | Dominated |  |  |
| *Group HN, Unknown FHx* | | | | | | | | | |
| CRA | 6158 | 312 | 6470 |  | 19.466 |  |  |  |  |
| PRS | 6093 | 418 | 6511 | 41 | 19.464 | -0.002 | Dominated |  |  |
| QRF&PRS | 6078 | 461 | 6539 | 69 | 19.466 | 0.000 | Dominated |  |  |
| QRF | 6170 | 422 | 6592 | 122 | 19.467 | 0.001 | 118994 |  |  |
| *Group HN, BC FHx* | | | | | | | | | |
| CRA | 6691 | 312 | 7003 |  | 19.351 |  |  |  |  |
| PRS | 6684 | 418 | 7102 | 100 | 19.351 | 0.000 | Dominated |  |  |
| QRF | 6703 | 422 | 7124 | 121 | 19.352 | 0.001 | 122874 |  |  |
| QRF&PRS | 6699 | 461 | 7160 | 158 | 19.353 | 0.002 | 38216 |  |  |
| *Group HN, OC FHx* | | | | | | | | | |
| QRF&PRS | 6874 | 461 | 7335 | -553 | 19.226 | -0.022 |  |  |  |
| PRS | 6939 | 418 | 7357 | -532 | 19.227 | -0.021 | 45115 |  |  |
| QRF | 7024 | 422 | 7446 | -442 | 19.227 | -0.021 | 247289 |  |  |
| CRA | 7576 | 312 | 7888 |  | 19.248 |  | 20948 |  |  |
| *Group HN, BCOC FHx* | | | | | | | | | |
| PRS | 7521 | 418 | 7939 | -467 | 19.250 | -0.022 |  |  |  |
| QRF&PRS | 7487 | 461 | 7949 | -458 | 19.250 | -0.022 | Dominated |  |  |
| QRF | 7549 | 422 | 7970 | -436 | 19.251 | -0.021 | 78416 |  |  |
| CRA | 8095 | 312 | 8407 |  | 19.272 |  | 20588 |  |  |
| *Group MH, Unknown FHx* | | | | | | | | | |
| QRF&PRS | 3758 | 461 | 4219 | -267 | 19.576 | -0.007 |  |  |  |
| QRF | 3809 | 422 | 4230 | -255 | 19.569 | -0.014 | Dominated |  |  |
| PRS | 3881 | 418 | 4299 | -187 | 19.579 | -0.004 | 27085 |  |  |
| CRA | 4174 | 312 | 4486 |  | 19.583 |  | 47373 |  |  |
| *Group MH, BC FHx* | | | | | | | | | |
| QRF&PRS | 4431 | 461 | 4892 | -301 | 19.516 | -0.033 |  |  |  |
| PRS | 4560 | 418 | 4978 | -219 | 19.521 | -0.028 | 17857 |  |  |
| QRF | 4684 | 422 | 5106 | -270 | 19.519 | -0.030 | Dominated |  |  |
| CRA | 5309 | 312 | 5621 |  | 19.549 |  | 22667 |  |  |
| *Group MH, OC FHx* | | | | | | | | | |
| CRA | 4712 | 312 | 5024 |  | 19.414 |  |  |  |  |
| QRF | 4686 | 422 | 5108 | 84 | 19.415 | 0.001 | 115513 |  |  |
| PRS | 4697 | 418 | 5116 | 92 | 19.423 | 0.009 | 1032 | 10796 |  |
| QRF&PRS | 4665 | 461 | 5126 | 103 | 19.422 | 0.008 | Dominated |  |  |
| *Group MH, BCOC FHx* | | | | | | | | | |
| PRS | 5400 | 418 | 5818 | -362 | 19.357 | -0.014 |  |  |  |
| QRF&PRS | 5360 | 461 | 5821 | -359 | 19.355 | -0.016 | Dominated |  |  |
| QRF | 5584 | 422 | 6006 | -174 | 19.358 | -0.013 | 296485 |  |  |
| CRA | 5868 | 312 | 6180 |  | 19.371 |  | 12922 | 25691 |  |
| *Group MN, Unknown FHx* | | | | | | | | | |
| CRA | 2193 | 312 | 2505 |  | 19.823 |  |  |  |  |
| PRS | 2200 | 418 | 2618 | 113 | 19.838 | 0.015 | 7550 |  |  |
| QRF | 2201 | 422 | 2623 | 118 | 19.828 | 0.005 | Dominated |  |  |
| QRF&PRS | 2198 | 461 | 2659 | 154 | 19.839 | 0.016 | 34060 |  |  |
| *Group MN, BC FHx* | | | | | | | | | |
| PRS | 2906 | 418 | 3325 | -290 | 19.777 | -0.015 |  |  |  |
| QRF&PRS | 2891 | 461 | 3352 | -263 | 19.776 | -0.016 | Dominated |  |  |
| QRF | 3124 | 422 | 3546 | -69 | 19.782 | -0.010 | 40090 |  |  |
| CRA | 3303 | 312 | 3615 |  | 19.792 |  | 6982 | 18822 |  |
| *Group MN, OC FHx* | | | | | | | | | |
| CRA | 2505 | 312 | 2817 |  | 19.741 |  |  |  |  |
| PRS | 2513 | 418 | 2931 | 114 | 19.755 | 0.014 | 7867 |  |  |
| QRF | 2514 | 422 | 2936 | 119 | 19.746 | 0.005 | Dominated |  |  |
| QRF&PRS | 2514 | 461 | 2975 | 157 | 19.757 | 0.016 | 32726 |  |  |
| *Group MN, BCOC FHx* | | | | | | | | | |
| PRS | 3217 | 418 | 3636 | -291 | 19.695 | -0.015 |  |  |  |
| QRF&PRS | 3203 | 461 | 3665 | -262 | 19.695 | -0.015 | Dominated |  |  |
| QRF | 3436 | 422 | 3857 | -69 | 19.700 | -0.009 | 42179 |  |  |
| CRA | 3614 | 312 | 3926 |  | 19.710 |  | 7291 | 19756 |  |
| *No PV, Unknown FHx* | | | | | | | | | |
| CRA | 993 | 312 | 1305 |  | 19.963 |  |  |  |  |
| QRF | 998 | 422 | 1419 | 114 | 19.963 | 0.001 | 214454 |  |  |
| PRS | 1014 | 418 | 1432 | 127 | 19.965 | 0.003 | 5871 | 47832 |  |
| QRF&PRS | 1018 | 461 | 1479 | 174 | 19.966 | 0.003 | 68986 |  |  |
| *No PV, BC FHx* | | | | | | | | | |
| CRA | 1631 | 312 | 1943 |  | 19.908 |  |  |  |  |
| PRS | 1525 | 418 | 1943 | 0 | 19.905 | -0.003 | Dominated |  |  |
| QRF | 1552 | 422 | 1974 | 31 | 19.905 | -0.003 | Dominated |  |  |
| QRF&PRS | 1530 | 461 | 1991 | 48 | 19.906 | -0.002 | Dominated |  |  |
| *No PV, OC FHx* | | | | | | | | | |
| CRA | 1313 | 312 | 1625 |  | 19.879 |  |  |  |  |
| QRF | 1318 | 422 | 1740 | 115 | 19.879 | 0.001 | 198293 |  |  |
| PRS | 1334 | 418 | 1752 | 127 | 19.881 | 0.003 | 5748 | 48299 |  |
| QRF&PRS | 1339 | 461 | 1800 | 175 | 19.882 | 0.003 | 59332 |  |  |
| *No PV, BCOC FHx* | | | | | | | | | |
| CRA | 1947 | 312 | 2259 |  | 19.825 |  |  |  |  |
| PRS | 1842 | 418 | 2260 | 1 | 19.822 | -0.003 | Dominated |  |  |
| QRF | 1869 | 422 | 2291 | 32 | 19.822 | -0.003 | Dominated |  |  |
| QRF&PRS | 1849 | 461 | 2310 | 51 | 19.824 | -0.002 | Dominated |  |  |

**Note: ICER was underlined if in the SW quadrant*

References:

1. Lee A, Mavaddat N, Wilcox AN, et al. BOADICEA: a comprehensive breast cancer risk prediction model incorporating genetic and nongenetic risk factors. *Genet Med.* 2019;21(8):1708-1718.

2. Lee A, Mavaddat N, Cunningham A, et al. Enhancing the BOADICEA cancer risk prediction model to incorporate new data on RAD51C, RAD51D, BARD1 updates to tumour pathology and cancer incidence. *J Med Genet.* 2022;59(12):1206-1218.

3. University of Cambridge. <https://www.canrisk.org/>. *CanRisk.* 2021.

4. Unit costs of health and social care. *Kent Academic Repository.* 2022.

5. Manchanda R, Patel S, Gordeev VS, et al. Cost-effectiveness of Population-Based BRCA1, BRCA2, RAD51C, RAD51D, BRIP1, PALB2 Mutation Testing in Unselected General Population Women. *J Natl Cancer Inst.* 2018;110(7):714-725.

6. National Health Service (NHS). *Adult Social Care Activity and Finance Report, England - 2020-21.*
