# Supplementary material for: Economic evaluation of personalised versus conventional risk assessment for women who have undergone testing for hereditary breast and ovarian cancer genes: a modelling study
Source: J Med Genet. 2025 Apr 10;62(7):e109948. doi: 10.1136/jmg-2024-109948 (PMC12322399; doi:10.1136/jmg-2024-109948)
Supplement: online supplemental file 2 [file jmg-62-7-s002.docx]

*Cost and health utility per patient by GPV status and family history*

|  | *Cost (UK£)* | | | | *QALY* | | *ICER* | | |
| --- | --- | --- | --- | --- | --- | --- | --- | --- | --- |
| Method* | *Cancer* | *Risk* | *Total* | *Δ* |  | *Δ* | *Full Incremental Analysis* | | |
| *Group HH, Unknown FHx* | | | | | | | | | |
| QRF&PRS | 8527 | 355 | 8882 | -34 | 19.091 | -0.006 |  |  |  |
| CRA | 8676 | 240 | 8916 |  | 19.097 |  | 5327 |  |  |
| QRF | 8603 | 324 | 8927 | 12 | 19.094 | -0.004 | Dominated |  |  |
| PRS | 8622 | 322 | 8944 | 28 | 19.095 | -0.002 | Dominated |  |  |
| *Group HH, BC FHx* | | | | | | | | | |
| QRF&PRS | 8967 | 355 | 9322 | -3 | 19.019 | -0.005 |  |  |  |
| CRA | 9085 | 240 | 9325 |  | 19.024 |  | 595 |  |  |
| QRF | 9015 | 324 | 9339 | 14 | 19.021 | -0.003 | Dominated |  |  |
| PRS | 9049 | 322 | 9370 | 46 | 19.022 | -0.002 | Dominated |  |  |
| *Group HH, OC FHx* | | | | | | | | | |
| CRA | 10302 | 240 | 10542 |  | 18.601 |  |  |  |  |
| PRS | 10290 | 322 | 10612 | 69 | 18.601 | 0.000 | Dominated |  |  |
| QRF | 10302 | 324 | 10626 | 84 | 18.601 | 0.000 | Dominated |  |  |
| QRF&PRS | 10278 | 355 | 10633 | 91 | 18.600 | -0.001 | Dominated |  |  |
| *Group HH, BCOC FHx* | | | | | | | | | |
| CRA | 11979 | 240 | 12219 |  | 18.139 |  |  |  |  |
| PRS | 11978 | 322 | 12300 | 81 | 18.138 | 0.000 | Dominated |  |  |
| QRF | 11979 | 324 | 12303 | 84 | 18.138 | 0.000 | Dominated |  |  |
| QRF&PRS | 11975 | 355 | 12330 | 110 | 18.138 | 0.000 | Dominated |  |  |
| *Group HN, Unknown FHx* | | | | | | | | | |
| CRA | 6158 | 240 | 6398 |  | 19.466 |  |  |  |  |
| PRS | 6093 | 322 | 6415 | 17 | 19.464 | -0.002 | Dominated |  |  |
| QRF&PRS | 6078 | 355 | 6432 | 34 | 19.466 | 0.000 | Dominated |  |  |
| QRF | 6170 | 324 | 6494 | 96 | 19.467 | 0.001 | 94183 |  |  |
| *Group HN, BC FHx* | | | | | | | | | |
| CRA | 6691 | 240 | 6931 |  | 19.351 |  |  |  |  |
| PRS | 6684 | 322 | 7006 | 75 | 19.351 | 0.000 | Dominated |  |  |
| QRF | 6703 | 324 | 7027 | 96 | 19.352 | 0.001 | 97228 |  |  |
| QRF&PRS | 6699 | 355 | 7054 | 123 | 19.353 | 0.002 | 28605 |  |  |
| *Group HN, OC FHx* | | | | | | | | | |
| QRF&PRS | 6874 | 355 | 7228 | -588 | 19.361 | -0.025 |  |  |  |
| PRS | 6939 | 322 | 7260 | -556 | 19.362 | -0.024 | 25002 |  |  |
| QRF | 7024 | 324 | 7349 | -467 | 19.364 | -0.022 | 48379 |  |  |
| CRA | 7576 | 240 | 7816 |  | 19.386 |  | 21184 |  |  |
| *Group HN, BCOC FHx* | | | | | | | | | |
| QRF&PRS | 7487 | 355 | 7842 | -493 | 19.250 | -0.022 |  |  |  |
| PRS | 7521 | 322 | 7843 | -492 | 19.250 | -0.022 | 1536 |  |  |
| QRF | 7549 | 324 | 7873 | -462 | 19.251 | -0.021 | 76327 |  |  |
| CRA | 8095 | 240 | 8335 |  | 19.272 |  | 21784 |  |  |
| *Group MH, Unknown FHx* | | | | | | | | | |
| QRF&PRS | 3758 | 355 | 4113 | -301 | 19.576 | -0.007 |  |  |  |
| QRF | 3809 | 324 | 4133 | -281 | 19.569 | -0.014 | Dominated |  |  |
| PRS | 3881 | 322 | 4202 | -212 | 19.579 | -0.004 | 30453 |  |  |
| CRA | 4174 | 240 | 4414 |  | 19.583 |  | 53585 |  |  |
| *Group MH, BC FHx* | | | | | | | | | |
| QRF&PRS | 4431 | 355 | 4786 | -763 | 19.516 | -0.033 |  |  |  |
| PRS | 4560 | 322 | 4882 | -667 | 19.521 | -0.028 | 19910 |  |  |
| QRF | 4684 | 324 | 5009 | -540 | 19.519 | -0.030 | Dominated |  |  |
| CRA | 5309 | 240 | 5549 |  | 19.549 |  | 23532 |  |  |
| *Group MH, OC FHx* | | | | | | | | | |
| CRA | 4712 | 240 | 4952 |  | 19.414 |  |  |  |  |
| QRF | 4686 | 324 | 5010 | 59 | 19.415 | 0.001 | 80594 |  |  |
| PRS | 4697 | 322 | 5019 | 67 | 19.423 | 0.009 | 1138 | 7915 |  |
| QRF&PRS | 4665 | 355 | 5020 | 68 | 19.422 | 0.008 | Dominated |  |  |
| *Group MH, BCOC FHx* | | | | | | | | | |
| QRF&PRS | 5360 | 355 | 5715 | -393 | 19.355 | -0.016 |  |  |  |
| PRS | 5400 | 322 | 5721 | -387 | 19.357 | -0.014 | 2745 |  |  |
| QRF | 5584 | 324 | 5909 | -199 | 19.358 | -0.013 | 295185 |  |  |
| CRA | 5868 | 240 | 6108 |  | 19.371 |  | 14805 | 27431 |  |
| *Group MN, Unknown FHx* | | | | | | | | | |
| CRA | 2193 | 240 | 2433 |  | 19.823 |  |  |  |  |
| PRS | 2200 | 322 | 2521 | 88 | 19.838 | 0.015 | 5909 |  |  |
| QRF | 2201 | 324 | 2525 | 92 | 19.828 | 0.005 | Dominated |  |  |
| QRF&PRS | 2198 | 355 | 2553 | 120 | 19.839 | 0.016 | 25941 |  |  |
| *Group MN, BC FHx* | | | | | | | | | |
| PRS | 2906 | 322 | 3228 | -315 | 19.777 | -0.015 |  |  |  |
| QRF&PRS | 2891 | 355 | 3245 | -298 | 19.776 | -0.016 | Dominated |  |  |
| QRF | 3124 | 324 | 3449 | -95 | 19.782 | -0.010 | 39940 |  |  |
| CRA | 3303 | 240 | 3543 |  | 19.792 |  | 9539 | 20411 |  |
| *Group MN, OC FHx* | | | | | | | | | |
| CRA | 2505 | 240 | 2745 |  | 19.741 |  |  |  |  |
| PRS | 2513 | 322 | 2835 | 89 | 19.755 | 0.014 | 6174 |  |  |
| QRF | 2514 | 324 | 2839 | 93 | 19.746 | 0.005 | Dominated |  |  |
| QRF&PRS | 2514 | 355 | 2868 | 123 | 19.757 | 0.016 | 25268 |  |  |
| *Group MN, BCOC FHx* | | | | | | | | | |
| QRF&PRS | 5360 | 355 | 5715 | -393 | 19.355 | -0.016 |  |  |  |
| PRS | 5400 | 322 | 5721 | -387 | 19.357 | -0.014 | 2745 |  |  |
| QRF | 5584 | 324 | 5909 | -199 | 19.358 | -0.013 | 295185 |  |  |
| CRA | 5868 | 240 | 6108 |  | 19.371 |  | 14805 | 27431 |  |
| *No PV, Unknown FHx* | | | | | | | | | |
| CRA | 993 | 240 | 1233 |  | 19.963 |  |  |  |  |
| QRF | 998 | 324 | 1322 | 89 | 19.963 | 0.001 | 166887 |  |  |
| PRS | 1014 | 322 | 1335 | 102 | 19.965 | 0.003 | 6261 | 38574 |  |
| QRF&PRS | 1018 | 355 | 1372 | 139 | 19.966 | 0.003 | 54405 |  |  |
| *No PV, BC FHx* | | | | | | | | | |
| PRS | 1525 | 322 | 1847 | -24 | 19.905 | -0.003 |  |  |  |
| CRA | 1631 | 240 | 1871 |  | 19.908 |  | 7826 |  |  |
| QRF | 1552 | 324 | 1877 | 6 | 19.905 | -0.003 | Dominated |  |  |
| QRF&PRS | 1530 | 355 | 1885 | 14 | 19.906 | -0.002 | Dominated |  |  |
| *No PV, OC FHx* | | | | | | | | | |
| CRA | 1313 | 240 | 1553 |  | 19.879 |  |  |  |  |
| QRF | 1318 | 324 | 1643 | 90 | 19.879 | 0.001 | 154528 |  |  |
| PRS | 1334 | 322 | 1655 | 102 | 19.881 | 0.003 | 6152 | 38942 |  |
| QRF&PRS | 1339 | 355 | 1694 | 141 | 19.882 | 0.003 | 47255 |  |  |
| *No PV, BCOC FHx* | | | | | | | | | |
| PRS | 1842 | 322 | 2164 | -23 | 19.822 | -0.003 |  |  |  |
| CRA | 1947 | 240 | 2187 |  | 19.825 |  | 7514 |  |  |
| QRF | 1869 | 324 | 2194 | 7 | 19.822 | -0.003 | Dominated |  |  |
| QRF&PRS | 1849 | 355 | 2204 | 17 | 19.824 | -0.002 | Dominated |  |  |

**Note: The optimal options were* ***bold***
